# Supplementary material for: Learning curve and surgical outcome of robotic assisted colorectal surgery with ERAS program
Source: Sci Rep. 2022 Nov 29;12:20566. doi: 10.1038/s41598-022-24665-w (PMC9709162; doi:10.1038/s41598-022-24665-w)
Supplement: Supplementary file 1 — Supplementary Information 1. [file 41598_2022_24665_MOESM1_ESM.zip › LSC Raw data-Ñ[▒K/110/H_ñw╝╨░O▒Kñσ.pdf]

正式摘要(電子簽章：000000206626182)

W55 / W55 ☐ 英文標題(注意:無英文姓名-請補病人基本檔!) ☐【讀取】憑證

臺中榮民總醫院

出院病歷摘要

轉入號： 轉出號：

[redacted]

(8)流水編號	
(9)入院日期	109/12/22 (2020) 直腸外科 W55 - 241
(10)轉科日期	
(11)出院日期	109/12/31 (2020) 住院天數計 9 日

診斷

- 入院 :Sigmoid colon cancer
- 出院 :Sigmoid colon cancer s/p laparoscopic anterior resection on 2020/12/24

主訴

- bloody stool for about 3 weeks

病史

- This 77 years old male patient didn't have special past history. He recieved hemiorrhaphy for left inguinal hernia on 2010. He complained bloody stool since end of November. He recieved colonoscopy that sigmoid colon tumor was found 18cm from anal verge and biopsy report showed malignancy. Abdmonial CT showed tumor sized 4.9cm and invaded non-peritonealized pericolonic or perirectal tissues. There was no abnormal lymphadenopathy in retroperitoneum, mesentery or bil. pelvic wall. or distent metastasis. Thus, he was admitted for surgical treatment.

體檢發現

- CEA: 2.4(2020/12)-
  2020/12/7 COS: tumor 18cm fav
  12/14 abd CT: GGO+
  12/18:lung CT.
  12/25: PET
  - General appearance :
    - easy looking , conscious clear , without cardiopulmonary distress
    - Vital signs自動帶入date 體溫(BT) 脈搏(P) 呼吸(R) 血壓(BP) 身高(BH) 體重(BW) BMI
      20201222 36.5℃ 82次/分 18次/分 133/59mmHg 155.4cm 60.6kg 25.1
  - HEENT :
    - Eyes: no anemic conjunctiva, no icteric sclera, normal EOM
    - ENT: no otorrhea, no rhinorrhea, no erythematous mucosa, no injected tonsil, no deformity
  - Neck: supple, freely movable, trachea midline,no deformity, no engorged jugular vein , no palpable thyroid or lymph node enlargement
  - Chest:
    - Inspection: Grossly normal, symmetric and fully expansion, no accessory muscle used, no operation scar, no spider angiomata, no gynecomastia
    - Palpation: no palpable mass or nodule, no subcutaneous emphysema, no tenderness
    - Ausculation: clear breathing sound
  - Heart:
    - Percussion: normal heart size
    - Ausculation: regular heart beat, normal S1, S2, no gallop, no murmur
  - Abdomen:
    - Inspection: in
      - flat contour, symetrically, no scar, no stria, no dilated veins, no rashes
    - Ausculation: normoactive bowel sound, no abdominal bruits
    - palpation: soft, no muscle guarding or rigidity, no local tenderness, no rebounding pain, no palpable mass, no palpable pulsation
    - Percussion: no shifting dulllness, no hepatomegaly, no splenomagaly
  -

Peripheral pulsation :	CA	BA	RA	FA	PA	PTA	DPA
Right side :	++	++	++	++	++	++	++/+++
Left side :	++	++	++	++	++	++	++/+++

  - Genito-anus: no tenderness or palpable mass lesion or rectal shelf, normal external genitalia, normal anal tone, brown stool

CRS CONDITION

- Rectodigital examination: none
- Colonoscope report: S colon. ca 18 cm FAV s/p Bx. another polyp. tatoo.
- CEA: 2.37 ng/ml
- Imaging studies(Sono , CT of abdomen and chest Films):
  - 檢驗項目 :CT ABDOMEN-SURVEY 報告日期 :1091222
    主 訴 :
    Result: Finding: Imaging Report Form for Colorectal Carcinoma (AJCC 8th) 1. Imaging Date: 20201214 Imaging Modality, Imaging by CT scan Imaging Protocol: CT scan of whole abdomen after IV and oral contrast was performed and reveals: 2. Tumor location / Size Location: Sigmoid Size: Measurable: 4.9 cm (largest diameter) 3. Tumor invasion Tumor invades non-peritonealized pericolonic or perirectal tissues 4. Regional nodal metastasis No 5. Distant metastasis (In this study) Single organ 6. Other findings: > Segmental wall thickening and loss of haustration at sigmoid colon, Axi#70, compatible with sigmoid colon cancer. > Bilateral hepatic cysts. > Bilateral renal cysts. > Bilateral renal stones less than 6mm. > Gallbladder, pancreas, spleen appear not unusual. > No abnormal lymphadenopathy in retroperitoneum, mesentery or bil. pelvic wall. > Mild subpleural GGO at RLL, Axi#5. > Mild thickening of left inguinal canal posterior wall. > Old fracture at left 6th rib. > DJD change with osteophytes formation of L spine Impression: Sigmoid colon cancer.
-
  - Lung

手術日期及方法

- 手術日期及方法20201224 1. Laparoscopic anterior resection with end-to-end anastomosis

住院治療經過

- After admission, operation with laparoscopic anterior resection was done on 2020/12/24. We let him try water on POD2 and then liquid diet, semiliquid diet on POD5. Drainage tube was removed on POD7. Under the stable condition, he was discharged on 12/31.

合併症

- nil

一般檢查

- 累積報告 :

DATE	TIME	ALB	TP	BIL.T	BIL.D	ALKP	AST	ALT	LDH	CHOL	TG	HDL-C	LDL	UA	NH3	AMY
1091222	1205	4.3		0.47	0.11	97	16	12	156							
1091223	1236		6.7							224	146		160	6.0		
1091224	1726	3.7		0.67	0.13	67	26	14								

DATE	TIME	WBC	RBC	HGB	HCT	MCV	PLT	BLST	NEUT	BAND	SEG	LYM	MON	EOS	BAS
1091222	1205	4400	4.18	13.1	39.0	93.3	207		60.2			27.7	8.2	3.2	0.7

1091224	1722	10670		12.2	37.4		184		92.6			5.6	1.5	0.1	0.2
1091228	0930	7810		12.7	37.0		182		78.9			11.7	6.1	3.2	0.1

DATE	TIME	NA	K	CL	CA	BUN	CREAT	Mg	P	CRP	ESR	PCT	LIPASE	LACTATE
1091222	1205	140	3.9		8.6	17	1.31							
1091223	1236									0.266				
1091224	1726	141	4.4		7.8	14	1.24							
1091228	0941	138	4.2			14	1.10							

DATE	TIME	GLUCOSE	PAB	TRF	ZN	CU	KETONE	CK	CKMB	PT-P	PT-C	APTT-P	APTT-C	TRO-T
1091222	1205	97								9.8	10.3	26.5		

特殊檢查

病理報告

其它

出院時情況

- 3.改門診治療

出院指示

出院計劃

主治醫師 2062F 蔣鋒帆 / 住院醫師 2331F 陳凱祥

正式摘要(電子簽章：000000206626182)

W55 / W55

doctorRank:B
